# Transcanal Endoscopic Ear Surgery for Conductive Hearing Loss in Postoperative Ears: A Report of Two Cases

**DOI:** 10.1155/crot/7846152

**Published:** 2026-08-21

**Authors:** Hisashi Sugimoto, Manabu Inaba, Goki Nagasaki, Tomokazu Yoshizaki

**Affiliations:** ^1^ Department of Otolaryngology-Head and Neck Surgery, Kanazawa University Graduate School of Medical Science, Kanazawa, Japan, kanazawa-u.ac.jp

**Keywords:** conductive hearing loss, postoperative ears, TEES

## Abstract

**Background:**

Revision middle ear surgery for persistent conductive hearing loss after previous tympanoplasty or ossiculoplasty is technically demanding because of scar tissue, anatomical distortion, and the unpredictable status of the ossicular chain. Although transcanal endoscopic ear surgery (TEES) has been increasingly adopted for primary tympanoplasty and ossiculoplasty, its feasibility as the sole surgical modality in postoperative ears has rarely been reported.

**Case Presentation:**

We describe two adult patients (Case 1: a 63‐year‐old man and Case 2: a 72‐year‐old man) who presented with long‐standing conductive hearing loss in dry, well‐aerated postoperative ears following previous tympanoplasty performed at outside institutions. Preoperative four‐frequency pure‐tone average (PTA) air‐bone gaps (ABGs) ranged from 28.75 to 40.00 dB (mean, 34.38 dB) with preserved bone‐conduction (BC) thresholds. In both patients, TEES was performed exclusively through a minimal skin incision just above the tympanic membrane, without a retroauricular approach. Intraoperatively, fixation of the ossicular chain involved not only the previously placed columella but also the native ossicles, requiring complete release in each case. Type III tympanoplasty was performed in both cases**.** According to the IOOG SAMEO‐ATO framework, both procedures were classified as S2r A1 Mx Ex Ox Ax Tx Ost.

**Outcome:**

The mean four‐frequency PTA air‐conduction threshold improved from 66.25 dB preoperatively to 42.19 dB postoperatively (mean hearing gain, 24.07 dB), and the mean postoperative ABG was reduced to 10.32 dB (range, 8.75–11.88 dB). Both patients (100%) fulfilled the criteria for postoperative hearing improvement according to the 2010 guidelines of the Japan Otological Society. No intraoperative or postoperative complications—including facial nerve injury, sensorineural hearing loss, dysgeusia, or vertigo—were observed during the follow‐up period (mean, 18.5 months; range, 12–25 months).

**Conclusion:**

In carefully selected postoperative ears with a dry middle ear and good BC reserve, TEES may serve as a useful minimally invasive alternative to the conventional retroauricular microscopic approach, offering advantages in surgical visualization, operability, and reduced invasiveness. Larger studies are warranted to define optimal indications and long‐term outcomes.

## 1. Introduction

Transcanal endoscopic ear surgery (TEES) has emerged over the past decade as a minimally invasive alternative to traditional microscopic ear surgery [1]. Advances in high‐definition endoscopic imaging and the development of dedicated single‐handed instruments have allowed surgeons to take advantage of the wide‐angle, off‐axis view afforded by the endoscope, enabling detailed inspection of hidden recesses of the middle ear—such as the sinus tympani, facial recess, and epitympanum—without the need for extensive bone removal or a retroauricular incision [2, 3].

The established indications for TEES have expanded steadily from myringotomy and myringoplasty to include tympanoplasty for chronic otitis media [4], congenital middle ear malformations [5], acquired cholesteatoma, tympanosclerosis, and stapes surgery for otosclerosis [6]. Several recent studies have also demonstrated that endoscopic ossiculoplasty achieves audiological outcomes comparable to those of microscopic ossiculoplasty, with reduced postoperative pain and morbidity.

Nonetheless, the use of TEES as the exclusive surgical modality in postoperative ears—that is, ears that have already undergone tympanoplasty or ossiculoplasty—remains poorly characterized. Revision middle ear surgery is particularly challenging because of scar formation, thinning of the canal skin, distorted anatomy, possible dehiscence of the facial nerve or lateral semicircular canal, and unpredictable status of the previously placed columella and the native ossicular chain [7]. To our knowledge, only limited evidence exists regarding whether TEES alone can safely and effectively address conductive hearing loss in these previously operated ears.

We recently treated two adult patients with long‐standing conductive hearing loss following prior tympanoplasty at outside institutions. All presented with dry, well‐aerated middle ears, a large air‐bone gap (ABG), and preserved bone‐conduction (BC) thresholds—a clinical profile in which the principal therapeutic goal is restoration of hearing rather than control of disease or mastoid cavity problems. In each case, we elected to perform TEES exclusively, with a minimal skin incision just above the tympanic membrane.

The aim of this report is twofold: (1) to describe the audiometric outcomes and perioperative course of two patients undergoing TEES for revision tympanoplasty and (2) to propose a surgical concept—including patient selection criteria, technical considerations, and anatomical landmarks—that may guide otologists considering this minimally invasive approach in postoperative ears.

## 2. Case Presentation

**FIGURE 1:**
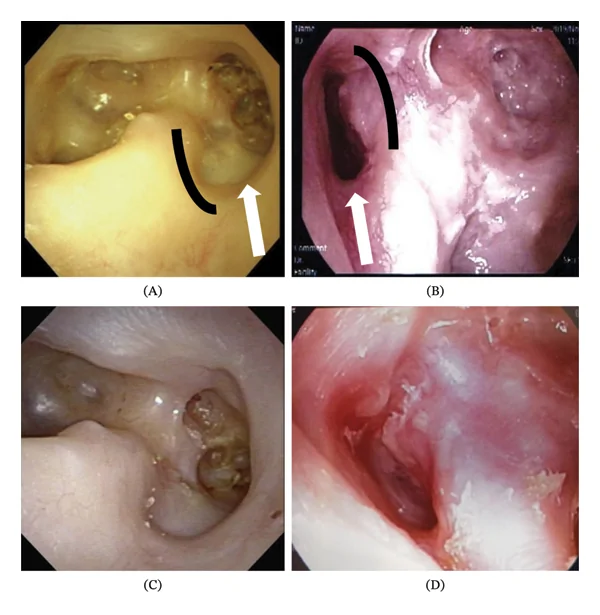
(A, B) Preoperative endoscopic findings in Cases 1 and 2. (C, D) Postoperative endoscopic findings in Cases 1 and 2. Black line: skin incision. The incision line is shown schematically to indicate the location of the skin incision just above the tympanic membrane and does not represent the exact incision length.

**FIGURE 2:**
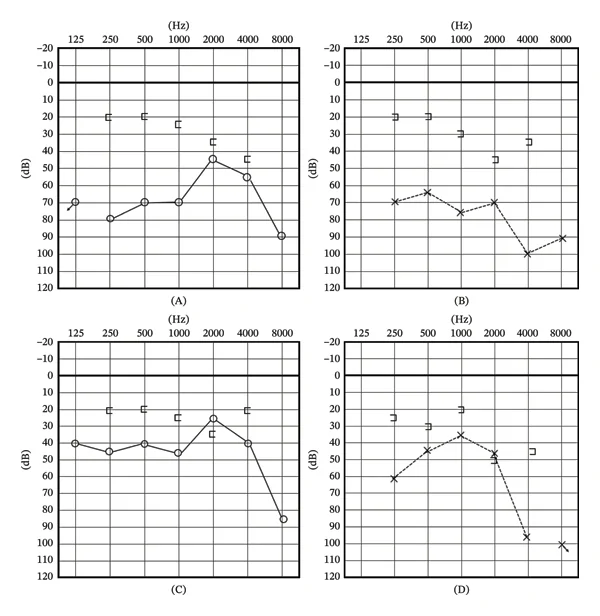
(A, B) Preoperative pure‐tone audiograms in Cases 1 and 2. (C, D) Postoperative pure‐tone audiograms in Cases 1 and 2.

**FIGURE 3:**
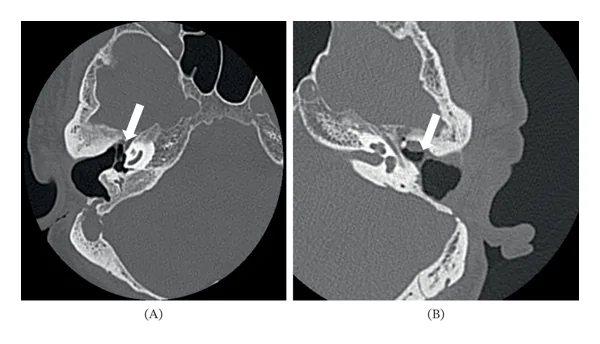
(A, B) Preoperative temporal bone computed tomography in Cases 1 and 2.

Two adult patients with postoperative ears presented with long‐standing conductive hearing loss. The patients were 63 and 72 years old at the time of surgery (Table 1). Both had previously undergone ear surgery at another hospital and had maintained dry ears without cavity problems for a prolonged period. Preoperative otoscopic/endoscopic examination showed stable postoperative ear findings in both cases (Figure 1A,B). Preoperative audiometry demonstrated good BC thresholds and a large ABG in both cases (Figure 2A,B). Temporal bone computed tomography confirmed preserved space in the tympanic cavity in each patient (Figure 3A,B). Based on these findings, TEES was selected as the sole surgical approach in both cases between November 2018 and April 2020. Before surgery, all patients underwent audiological evaluation. Pure‐tone thresholds were measured at 0.5, 1, 2, and 4 kHz, and the four‐frequency pure‐tone average (PTA) was calculated as (0.5 + 1 + 2 + 3) kHz/4, with the 3‐kHz threshold interpolated as the arithmetic mean of the 2‐ and 4‐kHz thresholds in accordance with the 1995 guidelines of the American Academy of Otolaryngology–Head and Neck Surgery (AAO‐HNS) [8]. Postoperative hearing outcomes were assessed according to the 2010 criteria of the Japan Otological Society, in which successful hearing improvement is defined as fulfilling at least one of the following: (1) a postoperative ABG of less than 15 dB, (2) a hearing improvement of more than 15 dB in air conduction (AC), or (3) a postoperative AC hearing level of less than 30 dB. Operative time and postoperative complications were also retrospectively reviewed. The tympanoplasty type was described according to the IOOG SAMEO‐ATO framework for standardized reporting of tympanomastoid surgery [9].

**TABLE 1:** Summary of patient demographics, intraoperative findings, and audiometric outcomes in two cases of transcanal endoscopic ear surgery for conductive hearing loss in postoperative ears.

Case	Age/sex	Tympanoplasty type	Operation time (min)	Preoperative			Postoperative			Hearing gain (dB)	JOS 2010 criteria met	Complications
				PTA‐AC (dB)	PTA‐BC (dB)	ABG (dB)	PTA‐AC (dB)	PTA‐BC (dB)	ABG (dB)			
1	63/M	Type III	141	58.75	30.00	28.75	35.63	26.88	8.75	23.13	(1), (2)	None
2	72/M	Type III	152	73.75	33.75	40.00	48.75	36.88	11.88	25.00	(1), (2)	None
Mean			146.5	66.25	31.88	34.38	42.19	31.88	10.32	24.07	2/2 (100%)	

*Note:* M, male; F, female. Four‐frequency pure‐tone average (PTA) was calculated as (0.5 + 1 + 2 + 3) kHz/4, with the 3‐kHz threshold interpolated as the arithmetic mean of the 2‐ and 4‐kHz thresholds, in accordance with the 1995 guidelines of the American Academy of Otolaryngology–Head and Neck Surgery. JOS 2010 criteria for postoperative hearing improvement: successful hearing improvement is defined as fulfilling at least one of the following: (1) a postoperative ABG of less than 15 dB; (2) a hearing improvement of more than 15 dB in air conduction; or (3) a postoperative AC hearing level of less than 30 dB. Hearing gain was calculated as preoperative PTA‐AC minus postoperative PTA‐AC.

Abbreviations: ABG, air‐bone gap; AC, air conduction; BC, bone conduction; JOS, Japan Otological Society; PTA, pure‐tone average.

### 2.1. Surgical Procedure

Intravenous antibiotics were administered preoperatively in all cases. After local infiltration with anesthetic and 1:100,000 epinephrine, a skin incision was made just above the tympanic membrane, and a minimal tympanomeatal flap was elevated (Figure 1A,B). In each case, fixation of the ossicular chain was released, including not only the previously reconstructed columella but also the native ossicles. No subcutaneous sutures were required. When middle ear structures were difficult to identify because of postoperative anatomical alteration, the cochleariform process was used as a landmark. All procedures were performed by a single surgeon.

### 2.2. Case 1

A 63‐year‐old man presented with long‐standing conductive hearing loss in the left ear. The patient had undergone tympanoplasty at another hospital several decades earlier; the exact date was not available. The ear had remained dry and free of cavity‐related problems for many years. Preoperative computed tomography demonstrated preserved middle ear space (Figure 3A). Preoperative pure‐tone audiometry revealed a four‐frequency PTA AC threshold of 58.75 dB and a BC threshold of 30.00 dB, yielding an ABG of 28.75 dB. Intraoperatively, the previous columella was identified as the incus. Fixation involved not only the reconstructed columella but also the native ossicles, whereas stapes mobility was preserved. After complete release of the fixation, Type III tympanoplasty was performed using the body of the incus. According to the IOOG SAMEO‐ATO framework, the procedure was classified as S_2_r A_1_ Mx Ex Ox Ax Tx Ost. Because the surgical objective was restoration of ossicular mobility and hearing, no additional tympanic membrane reinforcement, such as thinly sliced cartilage, was performed. The operative time was 141 min. At the latest follow‐up 12 months postoperatively, the PTA‐AC threshold had improved to 35.63 dB, with a postoperative ABG of 8.75 dB (hearing gain, 23.13 dB). This outcome fulfilled criteria (1) and (2) of the 2010 Japan Otological Society guidelines for postoperative hearing improvement. No deterioration of BC thresholds (postoperative BC, 26.88 dB) or other postoperative complications was observed.

### 2.3. Case 2

A 72‐year‐old man presented with long‐standing conductive hearing loss in the left ear, following tympanoplasty performed at another hospital 61 years earlier. The postoperative ear had remained dry and free of cavity‐related problems. Temporal bone computed tomography confirmed preserved tympanic cavity space (Figure 3B). Preoperative pure‐tone audiometry showed a four‐frequency PTA‐AC threshold of 73.75 dB and a PTA‐BC threshold of 33.75 dB, with an ABG of 40.00 dB. Intraoperatively, the previous columella was identified as the incus. The columella and the head of the malleus were strongly adherent to the lateral wall and were, therefore, removed. After release of the fixation, Type III tympanoplasty was performed using the head of the malleus. According to the IOOG SAMEO‐ATO framework, the procedure was classified as S_2_r A_1_ Mx Ex Ox Ax Tx Ost. The operative time was 152 min. At the latest follow‐up 25 months postoperatively, the PTA‐AC threshold had improved to 48.75 dB, with a postoperative ABG of 11.88 dB (hearing gain, 25.00 dB). This outcome fulfilled criteria (1) and (2) of the 2010 Japan Otological Society guidelines for postoperative hearing improvement. No deterioration of BC thresholds (postoperative BC, 36.88 dB) or other intraoperative or postoperative complications was noted.

### 2.4. Follow‐Up and Outcomes

Postoperative endoscopic findings showed that the tympanic membrane and external auditory canal in both patients were almost unchanged compared with their preoperative appearance (Figure 1C,D). Postoperative hearing improved in both cases, and both patients met the criteria for hearing improvement according to the 2010 Japan Otological Society guidelines (Table 1). The mean operative duration was 146.5 min. Postoperative complications, including facial nerve injury, sensorineural hearing loss, or vertigo, were not observed in any patient during the follow‐up period of 12–25 months (Table 1). No postoperative dysgeusia was observed clinically; however, the chorda tympani nerve was not clearly identified intraoperatively.

## 3. Discussion

The present report describes two patients with long‐standing conductive hearing loss in previously operated ears who were successfully managed using TEES as the sole surgical approach. In both cases, the middle ear was dry and BC thresholds were favorable, indicating that the principal therapeutic goal was restoration of hearing rather than control of active disease, recurrent cholesteatoma, or mastoid cavity problems. Under these conditions, both patients achieved postoperative hearing improvement without perioperative complications, suggesting that TEES may be a useful minimally invasive option in carefully selected postoperative ears.

Revision middle ear surgery is often technically demanding because postoperative ears may exhibit scar formation, thinning of the canal skin, altered anatomical landmarks, ossicular fixation, and potential exposure of critical structures such as the facial nerve or lateral semicircular canal. For these reasons, revision procedures have traditionally been performed through a retroauricular microscopic approach to secure a wide operative field. However, the transcanal endoscopic approach offers several theoretical and practical advantages in selected cases. Because the endoscope provides a wide‐angle, close‐up view with illumination at the tip, it can improve visualization of recesses and hidden middle ear structures that are difficult to inspect with the microscope’s linear line of sight. At the same time, avoiding a retroauricular incision may reduce surgical invasiveness and postoperative pain. A systematic review of endoscopic middle ear surgery has highlighted the superior visualization afforded by endoscopes, while also emphasizing that high‐quality comparative outcome data remain limited. More recent comparative studies have shown that endoscopic ossiculoplasty can achieve hearing outcomes comparable to those of microscopic surgery, with significantly shorter operative times, and meta‐analytic evidence suggests that endoscopic otologic surgery is associated with less postoperative pain than microscopic surgery [10–12].

### 3.1. Treatment Strategy

**FIGURE 4:**
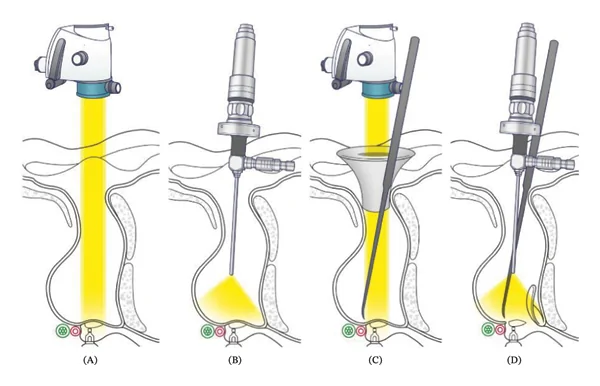
(A) Microscopic field of view. (B) Endoscopic field of view. (C) Microscopic procedure under otoscope. (D) Endoscopic technique. Minimal skin incision just above the eardrum. Red: facial nerve. Green: lateral semicircular canal.

In revision surgery for postoperative ears, the choice of surgical approach and instruments is of central importance. The available approaches include transcanal and retroauricular routes, while the principal optical systems are the microscope and the endoscope. In selected cases, the transcanal approach appears advantageous because it avoids a postauricular skin incision and may, therefore, reduce surgical invasiveness, postoperative pain, and operative duration. When a transcanal route is selected, the relative merits of the endoscope and microscope must be considered carefully. In our view, the endoscope offers important advantages in terms of surgical visualization. Microsurgery is inherently limited by a straight, linear field of view and cannot provide the same wide‐angle perspective; moreover, the observation point remains farther from the surgical target (Figure 4A). By contrast, the endoscope can be advanced close to the target, allowing detailed inspection of structures that would, otherwise, remain hidden from the microscopic line of sight while simultaneously providing a broad surgical overview (Figure 4B). From the standpoint of operability, microscopic transcanal procedures are often performed through an otoscope, and in many cases, this restricts the ability to use both hands freely while also reducing the available working space (Figure 4C). In contrast, endoscopic surgery provides operability comparable to that of conventional TEES. Taken together, these considerations suggest that TEES may offer superior visualization and favorable operability in selected postoperative ears.

### 3.2. Pathophysiological Implications of the Two Cases

The two cases in the present series illustrate different pathological mechanisms of conductive hearing loss in postoperative ears and expand the potential applicability of TEES in revision settings. In Case 1, hearing loss appeared to be primarily related to the status of the previous incus columella despite preserved stapes mobility and Type III tympanoplasty using the incus body provided functional improvement. This case suggests that TEES can be effective when the principal issue is reconstructive optimization in the presence of an, otherwise, mobile stapes. In Case 2, strong adhesions between the previous columella, the malleus head, and the lateral wall represented a different mechanism—ossicular fixation caused by postoperative adhesion. Release of fixation and reconstruction with the malleus head resulted in hearing improvement, emphasizing the importance of identifying and addressing not only the previously reconstructed columella but also fixation involving native ossicles.

### 3.3. Indications

These cases also provide practical insight into the indications for TEES in postoperative ears. In our opinion, TEES should be considered primarily in patients with a dry and stable middle ear, preserved tympanic cavity aeration, and favorable BC reserve. In such situations, the main objective of surgery is hearing restoration rather than management of cavity problems or recurrent disease. By contrast, in patients with active cavity problems, poor aeration, recurrent pathology, or disease extending beyond the area that can be safely managed transcanally, a conventional microscopic approach with a retroauricular incision may remain more appropriate [7].

TEES should, therefore, not be regarded as universally applicable to all postoperative ears. Given the very small number of cases in the present series, no definitive conclusion can yet be drawn regarding precise indications. Further accumulation of cases is needed to clarify the factors associated with successful outcomes. In addition, patients should be informed preoperatively that surgery may initially be exploratory in nature. If intraoperative findings reveal a surgically correctable pathology, definitive reconstruction can proceed; if not, the procedure may need to be limited. Patients should also be counseled that favorable hearing results cannot be guaranteed in every case.

### 3.4. Surgical Techniques

The present cases underscore several important technical considerations. First, we used only a minimal skin incision just above the tympanic membrane (Figure 4D) because the skin in postoperative ears is often thin and flap elevation can be technically difficult and potentially hazardous. Limiting flap elevation may help avoid injury to critical structures such as the facial nerve and the lateral semicircular canal, both of which may already be exposed as a consequence of previous surgery. A larger tympanomeatal flap in such ears could increase the risk of damaging these vulnerable structures. Second, the complete release of ossicular fixation is essential. In postoperative ears, fixation commonly involves not only the previously inserted columella but also residual native ossicles, and failure to release these adhesions adequately may compromise hearing restoration. Third, when familiar anatomical landmarks are obscured by prior surgery, the cochleariform process may serve as a useful and reliable landmark for orientation. Because this robust structure is often preserved even after multiple previous operations, it can facilitate identification of the facial nerve and other critical middle ear structures.

Taken together, these cases support several practical considerations for patient selection and surgical planning. TEES should probably be reserved for postoperative ears with a dry and stable middle ear, preserved tympanic cavity aeration, and good BC reserve. At the same time, the success of this approach depends not only on case selection but also on meticulous surgical execution, including minimal flap elevation, careful identification of landmarks, and complete release of ossicular fixation. These concepts are consistent with prior endoscopic literature emphasizing the strengths of transcanal visualization, but they also reflect the need for careful case selection rather than indiscriminate application of TEES to all revision ears [10].

### 3.5. Limitations

The present study has several limitations. First, this was a retrospective report of only two cases, and the sample size is clearly insufficient to define firm indications or to generalize the results. Second, the cases were highly selected, as all patients had dry ears, preserved middle ear space, and favorable BC; therefore, the results should not be extrapolated to all postoperative ears. Third, the absence of a formal control group prevents robust comparison with microscopic retroauricular surgery, particularly with regard to operative time, which may be influenced by case selection and other background factors. Fourth, detailed long‐term follow‐up data, including stability of hearing gain and recurrence of ossicular fixation, were limited. Finally, while TEES provides excellent visualization, one‐handed surgery remains an inherent technical limitation of the endoscopic technique and may become problematic in cases with bleeding or more extensive disease. Accordingly, the current findings should be interpreted as hypothesis‐generating rather than definitive.

## 4. Conclusion

In conclusion, our experience suggests that TEES can be an effective and minimally invasive option for selected postoperative ears with conductive hearing loss when the objective is hearing restoration in a dry, well‐aerated middle ear. The present two cases demonstrate that the technique may be applicable in selected revision Type III tympanoplasty cases, provided that careful patient selection, meticulous release of ossicular fixation, and appropriate reconstruction are achieved. Larger comparative studies with standardized audiometric and long‐term outcome assessment are needed to clarify the role of TEES in revision middle ear surgery.

## Author Contributions

Hisashi Sugimoto: conceptualization, methodology, investigation, surgery, and writing–original draft.

Manabu Inaba: supervision and writing–review and editing.

Goki Nagasaki: data curation and investigation.

Tomokazu Yoshizaki: supervision and project administration.

## Funding

This research did not receive any specific grant from funding agencies.

## Ethics Statement

This study was conducted in accordance with the Declaration of Helsinki. The Institutional Review Board of Kanazawa University determined that ethical approval was not required for this single case report. Written informed consent for publication, including clinical data and images, was obtained from the patient.

## Conflicts of Interest

The authors declare no conflicts of interest.

## Supporting Information

Additional supporting information can be found online in the Supporting Information section.

## Supporting information


**Supporting Information** CARE Checklist publication.

## Data Availability

The data are not publicly available due to patient privacy concerns but may be available from the corresponding author upon reasonable request.

## References

[1] Tarabichi M. , Endoscopic Middle Ear Surgery, Annals of Otology, Rhinology & Laryngology. (1999) 108, no. 1, 39–46, 10.1177/000348949910800106. 9930539

[2] Cohen M. S. , Landegger L. D. , Kozin E. D. , and Lee D. J. , Pediatric Endoscopic Ear Surgery in Clinical Practice: Lessons Learned and Early Outcomes, The Laryngoscope. (2016) 126, no. 3, 732–738, 10.1002/lary.25410. 26228434

[3] Furukawa T. , Watanabe T. , Ito T. , Kubota T. , and Kakehata S. , Feasibility and Advantages of Transcanal Endoscopic Myringoplasty, Otology & Neurotology. (2014) 35, no. 4, e140–e145, 10.1097/mao.0000000000000298. 24622030

[4] Ayache S. , Cartilaginous Myringoplasty: the Endoscopic Transcanal Procedure, European Archives of Oto-Rhino-Laryngology. (2013) 270, no. 3, 853–860, 10.1007/s00405-012-2056-x. 22639200

[5] Ito T. , Kubota T. , Furukawa T. , Matsui H. , Futai K. , and Kakehata S. , Transcanal Endoscopic Ear Surgery for Congenital Middle Ear Anomalies, Otology & Neurotology. (2019) 40, no. 10, 1299–1305, 10.1097/mao.0000000000002393. 31634283

[6] Kojima H. , Komori M. , Chikazawa S. et al., Comparison Between Endoscopic and Microscopic Stapes Surgery, The Laryngoscope. (2014) 124, no. 1, 266–271, 10.1002/lary.24144. 23670854

[7] Kinney S. E. , The Problem Mastoid Cavity: Medical and Surgical Management, The Laryngoscope. (1977) 87, no. 11, 1853–1860, 10.1002/lary.1977.87.11.1853. 916779

[8] Committee on Hearing and Equilibrium Guidelines for the Evaluation of Results of Treatment of Conductive Hearing Loss. AmericanAcademy of Otolaryngology-Head and Neck Surgery Ffoundation, Otolaryngology–Head and Neck Surgery. (1995) 113, no. 3, 186–187. 7675477 10.1016/S0194-5998(95)70103-6

[9] Yung M. , James A. , Philips J. et al., International Otology Outcome Group and the International Consensus on the Categorization of Tympanomastoid Surgery, Journal of International Advanced Otology. (2018) 14, no. 2, 216–226, 10.5152/iao.2018.5553. 30100547 PMC6354466

[10] Kozin E. D. , Gulati S. , Kaplan A. B. et al., Systematic Review of Outcomes Following Observational and Operative Endoscopic Middle Ear Surgery, The Laryngoscope. (2015) 125, no. 5, 1205–1214, 10.1002/lary.25048. 25418475 PMC4467784

[11] Celik O. and Ulkumen B. , Endoscopic Versus Microscopic Ossiculoplasty: Does the Functional Outcome Vary According to the Type of Osciculoplasty?, Brazilian Journal of Otorhinolaryngology. (2023) 89, no. 2, 213–221, 10.1016/j.bjorl.2022.02.003. 35428604 PMC10071538

[12] Toulouie S. , Block-Wheeler N. R. , and Rivero A. , Postoperative Pain After Endoscopic vs Microscopic Otologic Surgery: a Systematic Review and Meta-Analysis, Otolaryngology–Head and Neck Surgery. (2022) 167, no. 1, 25–34, 10.1177/01945998211041946. 34491858

