# Supplementary material for: Transcanal Endoscopic Ear Surgery for Conductive Hearing Loss in Postoperative Ears: A Report of Two Cases
Source: Case Rep Otolaryngol. 2026 Aug 21;2026:7846152. doi: 10.1155/crot/7846152 (PMC13495555; doi:10.1155/crot/7846152)
Supplement: Supplementary file 1 [file CROT-2026-7846152-s001.docx]

**CARE Checklist**

1. Title Identify the diagnosis or intervention of primary focus and indicate that this is a case report Title Included: “A Report of Two Cases”

2. Keywords 2–5 key words describing the main diagnosis/intervention, including “case report” if possible Keywords Suggested: TEES; postoperative ears; conductive hearing loss; revision tympanoplasty; case report

3. Abstract Summarize the case(s), main findings, intervention, outcomes, and key lesson(s) Abstract Structured abstract present: Background / Case Presentation / Outcome / Conclusion

4. Introduction Brief background and rationale for reporting the case(s) Introduction Included: background of TEES, challenges of revision middle ear surgery, rationale for the report

5. Patient Information De-identified demographic details, main concerns, medical/surgical history, and relevant previous interventions Case Presentation – Case 1 / Case 2 Included: age, sex, previous tympanoplasty at another hospital, long-standing conductive hearing loss

6. Clinical Findings Important clinical and physical examination findings Case Presentation Included: dry postoperative ears, preserved middle ear aeration, favorable bone conduction, enlarged ABG

7. Timeline Historical and current information organized as a timeline Not separately presented Not fully included as an independent table/figure; optional but recommended

8. Diagnostic Assessment Diagnostic methods, tests, imaging, and assessment process Methods / Case Presentation Included: pure-tone audiometry, PTA-AC, PTA-BC, ABG, temporal bone CT, AAO-HNS 1995 guideline, JOS 2010 criteria

9. Therapeutic Intervention Details of the intervention(s), including procedure and technique Surgical Procedure / Case Presentation Included: TEES, minimal skin incision above the tympanic membrane, release of ossicular fixation, type III tympanoplasty

10. Follow-up and Outcomes Clinical course after intervention, follow-up findings, outcomes, and adverse events Outcome / Postoperative Course Included: hearing improvement, postoperative PTA-AC and ABG, no complications, follow-up period

11. Discussion Strengths, limitations, interpretation, relevant literature, and take-away message Discussion Included: advantages of TEES, indications, technical points, comparison with prior literature, limitations

12. Patient Perspective Patient-reported experience or perception of the treatment Not separately presented Not currently included; optional but recommended if available

13. Informed Consent Confirmation that informed consent for publication was obtained Ethics Statement / Consent Included: written informed consent for publication obtained
